# Mutational landscape of cancer-driver genes across human cancers

**DOI:** 10.1038/s41598-023-39608-2

**Published:** 2023-08-07

**Authors:** Musalula Sinkala

**Affiliations:** 1 Department of Biomedical Sciences, School of Health Sciences, University of Zambia, Lusaka, Zambia; 2 Computational Biology Division, Faculty of Health Sciences, Institute of Infectious Disease and Molecular Medicine, University of Cape Town, Cape Town, South Africa

**Keywords:** Cancer genomics, Data integration, Data mining, Genome informatics, Cancer genetics, Mutation

## Abstract

The genetic mutations that contribute to the transformation of healthy cells into cancerous cells have been the subject of extensive research. The molecular aberrations that lead to cancer development are often characterised by gain-of-function or loss-of-function mutations in a variety of oncogenes and tumour suppressor genes. In this study, we investigate the genomic sequences of 20,331 primary tumours representing 41 distinct human cancer types to identify and catalogue the driver mutations present in 727 known cancer genes. Our findings reveal significant variations in the frequency of cancer gene mutations across different cancer types and highlight the frequent involvement of tumour suppressor genes (94%), oncogenes (93%), transcription factors (72%), kinases (64%), cell surface receptors (63%), and phosphatases (22%), in cancer. Additionally, our analysis reveals that cancer gene mutations are predominantly co-occurring rather than exclusive in all types of cancer. Notably, we discover that patients with tumours displaying different combinations of gene mutation patterns tend to exhibit variable survival outcomes. These findings provide new insights into the genetic landscape of cancer and bring us closer to a comprehensive understanding of the underlying mechanisms driving the development of various forms of cancer.

## Introduction

Cancer is a complex disease characterised by mutations in genes that control various hallmarks of the disease, including escaping programmed cell death, promoting genome instability and mutations, and proliferative signalling^1^. Cancer genes include genes encoding cell surface receptors, oncogenes, tumour suppressor genes, kinases, phosphatases, and transcription factors^2–6^. Cancer genes of these classes transcribe mRNAs that encode proteins, which function in various oncogenic pathways that fuel oncogenesis by enabling various hallmarks of cancer^7^. The mutations in known cancer genes, unlike those in non-cancer driver genes^8–10^, qualitatively or quantitatively alter the function of genes and proteins and, consequently, alter the cellular processes in which these proteins participate. Moreover, oncogene mutations are linked with differences in patient survival, clinical outcomes, metastatic or recurrent tumours, and serve as predictors of tumour responsiveness to anti-cancer drugs^8,9,11,12^. There is, therefore, a need to understand the extent to which cancer genes are mutated in cancers of different tissues of origin.

Over the last few decades, our understanding of the genes, pathways, and their role in oncogenesis has grown significantly, leading to increased efforts to treat various cancer types^13–18^. Furthermore, many genetic aberrations have been identified in human cancers, and several of the proteins encoded by these genes are well-established drug targets, while others are promising drug targets^3,4,19,20^. However, despite this impressive list of known gene mutations, it covers only a few cancer types. To comprehend the extent and consequences of gene alterations affecting function, it is crucial to study the alteration of cancer genes across all human cancers and within each cancer category. Such knowledge has been successfully applied in the design of therapies explicitly targeting proteins altered by somatic and germline mutations in cancer genes^21,22^. However, we still do not completely understand the extent to which cancer genes and the classes thereof are altered in all human cancers.

Here, we utilise publicly available datasets generated by various cancer sequencing projects to understand the extent of cancer gene alterations in human cancers. Furthermore, we obtain information on known cancer genes compiled by the Catalog of Somatic Mutations in Cancer (COSMIC), Cancer Gene Consensus (CGC) database^23,24^. Then, we comprehensively analyse known cancer gene mutations across different cancer types by integrating information on tumour genetic alterations with known gene annotations. Our analysis provides novel biological insights into the mutational landscape of these cancer genes and shows the extent to which they co-occur or are exclusive in tumours of various tissues and their association with patient outcomes.

## Results

### Mutational landscape of cancer genes in human cancers

Our study compiled a list of 727 known cancer genes based on information from the Catalogue of Somatic Mutations in Cancer (COSMIC)^24^ and Cancer Gene Consensus (CGC)^23^ database. This list includes genes that encode oncogenes (383), tumour suppressor genes (TSGs; 370), transcription factors (150), kinases (75), phosphatases (9), and cell surface receptor proteins (CSR; 63) (see Supplementary Fig. 1, and Supplementary Data 1).

**Figure 1:**
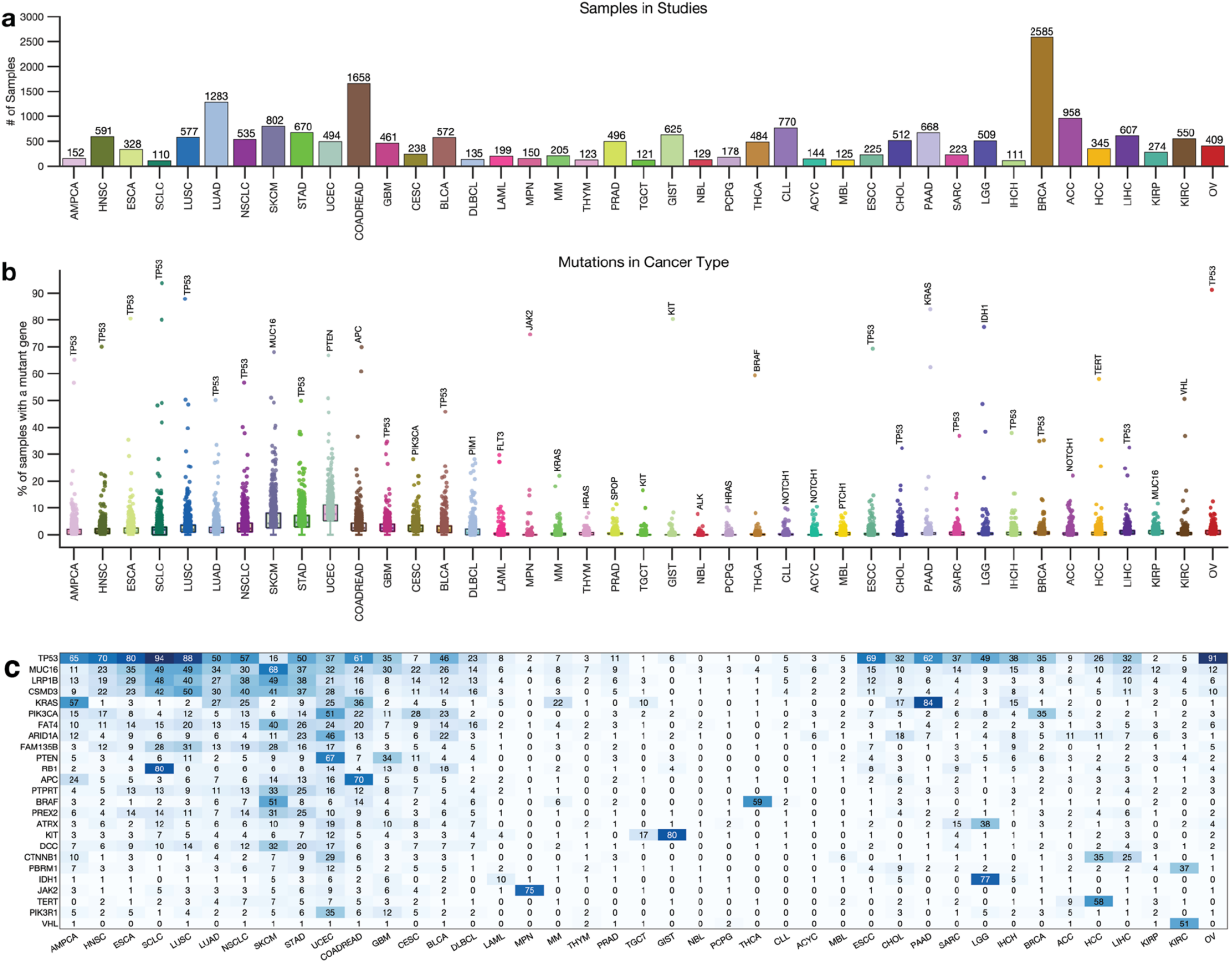
Distribution of cancer types and known cancer gene mutations in 20,066 tumours. (**a**) Distribution of 20,066 tumours across 43 human cancer types. The disease codes and abbreviations: UCEC, uterine corpus endometrial carcinoma; SKCM, skin cutaneous melanoma; BLCA, bladder urothelial carcinoma; UCS, uterine carcinosarcoma; OV, ovarian serous cystadenocarcinoma; LUSC, lung squamous cell carcinoma; STAD, stomach adenocarcinoma; LUAD, lung adenocarcinoma; ESCA, oesophageal adenocarcinoma; DLBC, diffuse large b-cell lymphoma; CESC, cervical squamous cell carcinoma; HNSC, head and neck squamous cell carcinoma; SARC, sarcoma; LIHC, liver hepatocellular carcinoma; BRCA, breast invasive carcinoma; COADREAD, colorectal adenocarcinoma; CHOL, cholangiocarcinoma; ACC, adrenocortical carcinoma; PAAD, pancreatic adenocarcinoma; PRAD, prostate adenocarcinoma; GBM, glioblastoma multiforme; KIRP, kidney renal papillary cell carcinoma; KIRC, kidney renal clear cell carcinoma; MESO, mesothelioma; LGG, brain lower grade glioma; UVM, uveal melanoma; PCPG, pheochromocytoma and paraganglioma; TGCT, testicular germ cell tumours; KICH, kidney chromophobe; THYM, thymoma; LAML, acute myeloid leukaemia; THCA, thyroid carcinoma. Panel (**b**) shows the mutation frequency of known cancer genes in each cancer type, and panel (**c**) is a clustered heatmap showing the most frequently mutated cancer genes across types, based on the percentage of tumours with altered genes. The heatmap was generated using unsupervised hierarchical clustering with the Cosine distance metric and complete linkage. The colour intensity increases to represent a higher percentage (see Supplementary Fig. 2).

Next, we obtained whole-exome sequencing data from 138 cancer studies focusing on 41 different human cancer types and involving 20,331 samples (Supplementary Data 1). The number of samples in each study varied, with breast carcinoma representing the highest number of samples (2,585) and Small-Cell Lung Cancer the least (110), as shown in Fig. 1a. We then calculated the somatic mutation frequencies in the 727 genes across the samples of the 41 cancer types (Supplementary Data 1) and found that the mutation frequency for different cancer genes ranged from 0% for the *VHL* (and other genes) in acute myeloid leukaemia to 94% for the *TP53* gene in small cell lung carcinomas (Fig. 1b,c). Other cancer genes with high mutation frequencies include *KRAS* (mutated in 84%) of pancreatic adenocarcinomas and *PTEN* (67%) of uterine corpus endometrial carcinomas, and *JAK2* (75%) of myeloproliferative neoplasms (Fig. 1c, also see Supplementary Data 1).

Next, for each cancer type, we summarised the number of mutated genes in (1) none of the samples, (2) less than 5 per cent of the samples, and (3) more than 5% of the samples. Our analysis revealed that most cancer genes were not mutated cancer types, and a limited number of genes were found to be mutated in over 5% of the samples. For instance, only two known cancer genes were found to be mutated in over 5% of thymomas (*MUC16* and *HRAS*), testicular germ cell tumours (*KRAS* and *KIT*), and thyroid carcinomas (*BRAF* and *NRAS*) (Fig. 1c, Supplementary Fig. 2, and Supplementary Data 1).

Furthermore, some cancer types had a significantly higher number of known cancer genes mutated in more than 5% of the samples, e.g., in uterine corpus endometrial carcinoma (568 known cancer genes mutated), stomach adenocarcinoma (330 genes), and skin cutaneous melanoma (314 genes). This finding shows that the extent to which the cancer genes are mutated across cancer types varies and that some cancer types have few mutations within the coding sequences of known cancer genes^25,26^.

### Mutations of each cancer gene across all tumours

**Figure 2:**
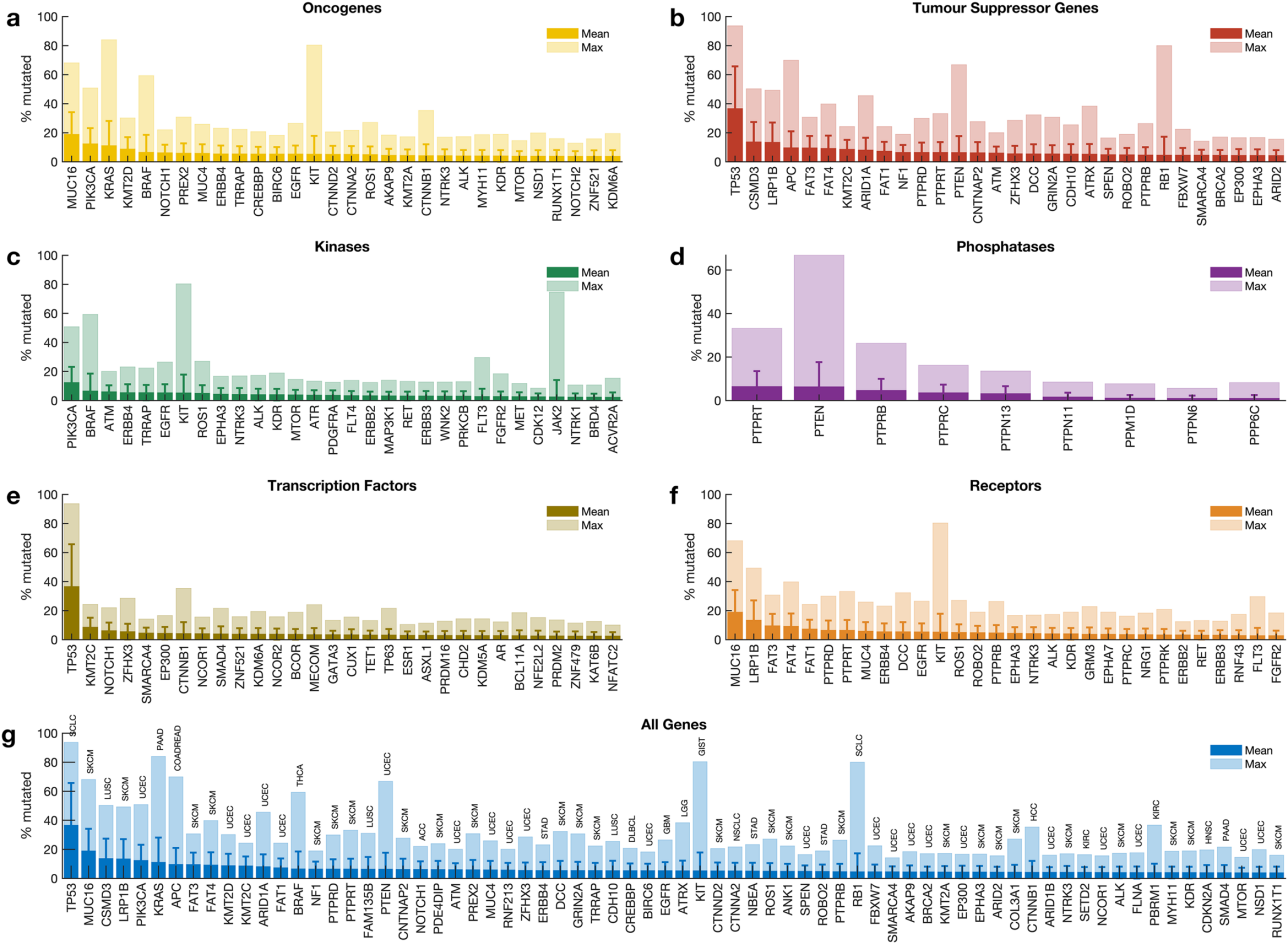
Frequency of cancer gene mutations in different gene classes. Frequency of known cancer gene mutations among: (**a**) oncogenes, (**b**) tumour suppressor genes, genes that encode (**c**) kinases, (**d**) phosphatases, (**e**) transcription factors, (**f**) cell surface receptor proteins, (**g**) all the genes.

We calculated the mutation frequency of each cancer gene across 41 different human cancer types in all 20,331 samples. Our analysis revealed that 98.9% (719 out of 727) of cancer genes were mutated in at least one sample. Among the oncogenes, *MUC16* (mutated in 18.9% of tumours), *PIK3CA* (12.4%), and *KRAS* (11.1%) were the most frequently mutated across the profiled samples (Fig. 2a). Furthermore, among the TSGs, *TP53* (36.6%), *CSMD3* (13.7%), and *LRP1B* (13.5%) were the most frequently mutated (Fig. 2b). *PIK3CA* (12.4%), *BRAF* (6.6%), and *ATM* (6.0%) were the top three mutated genes that encode protein kinases (Fig. 2c). In addition, we found that *PTPRT* (6.5%) and *PTEN* (6.4%) were the most frequently mutated among the genes that encode protein phosphatases (Fig. 2d), while *TP53* (36.6%) and *KMT2C* (8.6%) were the top-two frequently mutated among the genes that encode transcription factors (Fig. 2e). Furthermore, among the genes that encode cell surface receptors, we found that *MUC16* (18.9%) and *LRP1B* (13.5%) were the most frequently mutated (Fig. 2f). Overall, the top-five frequently mutated cancer genes across human cancers were *TP53* (36.6%), *MUC16* (18.9%), *CSMD3* (13.7%), *LRP1B* (13.5%), and *PIK3CA* (12.4%) (Fig. 2g).

The mutation frequencies we report here are reasonably consistent with previous reports, which indicated that *TP53* (36.6% across all samples) is the most frequently altered gene, followed by *PIK3CA* (12.4%)^5,27^. Furthermore, we found that the extent to which the cancer genes are mutated in different cancer types varies significantly, a pattern likely to impact the treatment strategies that could be applied to cancers of different tissues^28^.

### Mutations in categories of cancer genes

**Figure 3:**
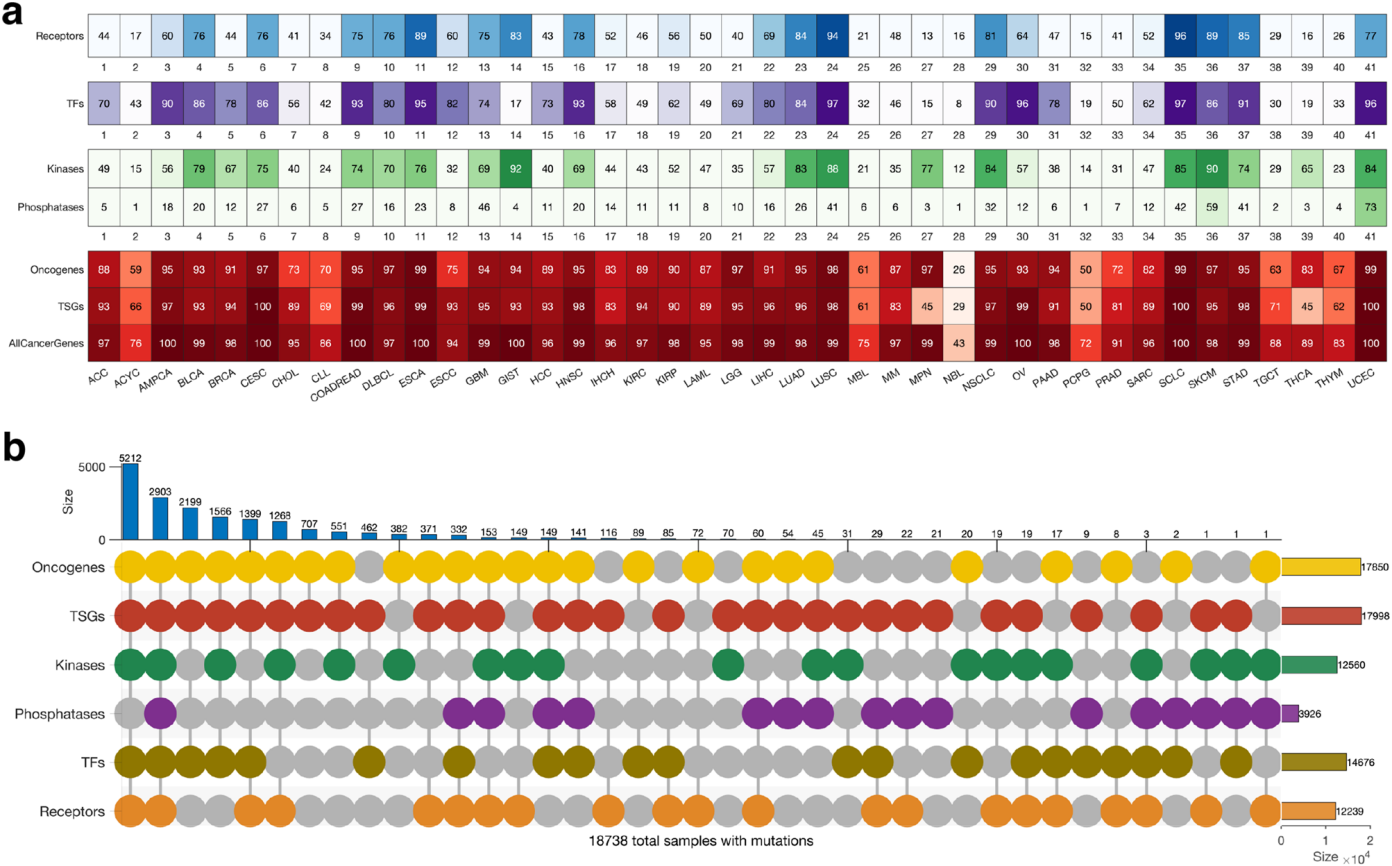
Gene alterations and mutational landscape in different cancer types. (**a**) The frequency of altered genes encoding cell surface receptors, transcription factors, kinases, phosphatases, oncogenes, tumour suppressor genes and all the cancer genes in different types of tumours. (**b**) A plot showing the mutual exclusivity and co-occurrence of mutations in the different classes of cancer genes, only considering mutations in tumours with mutations in genes that belong to more than one class. Refer to Supplementary Fig. 5 for the count of mutations exclusive to each class of genes.

We were interested in evaluating the extent to which genes in particular categories of cancer genes (oncogenes, TSGs, transcription factors, kinases, phosphatases, and receptors) are mutated across human cancers. Here, we found mutations in the known cancer genes in all tumours. Of the six classes of cancer genes, the TSGs (91% of the tumours) and the oncogenes (89%) showed the highest frequency of mutations, followed by the transcription factors (72%), kinases (62%), receptors (60%), and the phosphatases (19%); (Supplementary Fig. 3 and Fig. 3a). Overall, our analyses revealed that the mutational landscape of the six cancer gene classes was mainly consistent within cancer (Fig. 3a). Therefore, we suggest that the observed correlation in mutation frequencies between cancer genes of different classes in a particular cancer type may indicate that gene mutations tend to co-occur (see Supplementary Fig. 4).

Also, we found that 92% (18,738 samples) of all the tumours harboured mutations in genes involved in more than one of the six cancer gene classes (Fig. 3b). Furthermore, we found 2,903 tumours harbouring mutations in all six classes of genes, and another 5,212 tumours harbouring mutations in all six classes of genes, except those that encode protein phosphatases (Fig. 3b). Conversely, among all the cancer types, we found that 737 samples harboured mutations in only one class of the known cancer genes (Supplementary Fig. 5). The percentage of mutated cancer genes that are members of multiple cancer gene categories is shown in Supplementary Fig. 6. Overall, our findings demonstrate that for most cancer types, the tumours tend to have mutations in the genes of at least five of the six classes of cancer genes.

### Co-occurrence and exclusivity of mutations in cancer gene pairs

**Figure 4:**
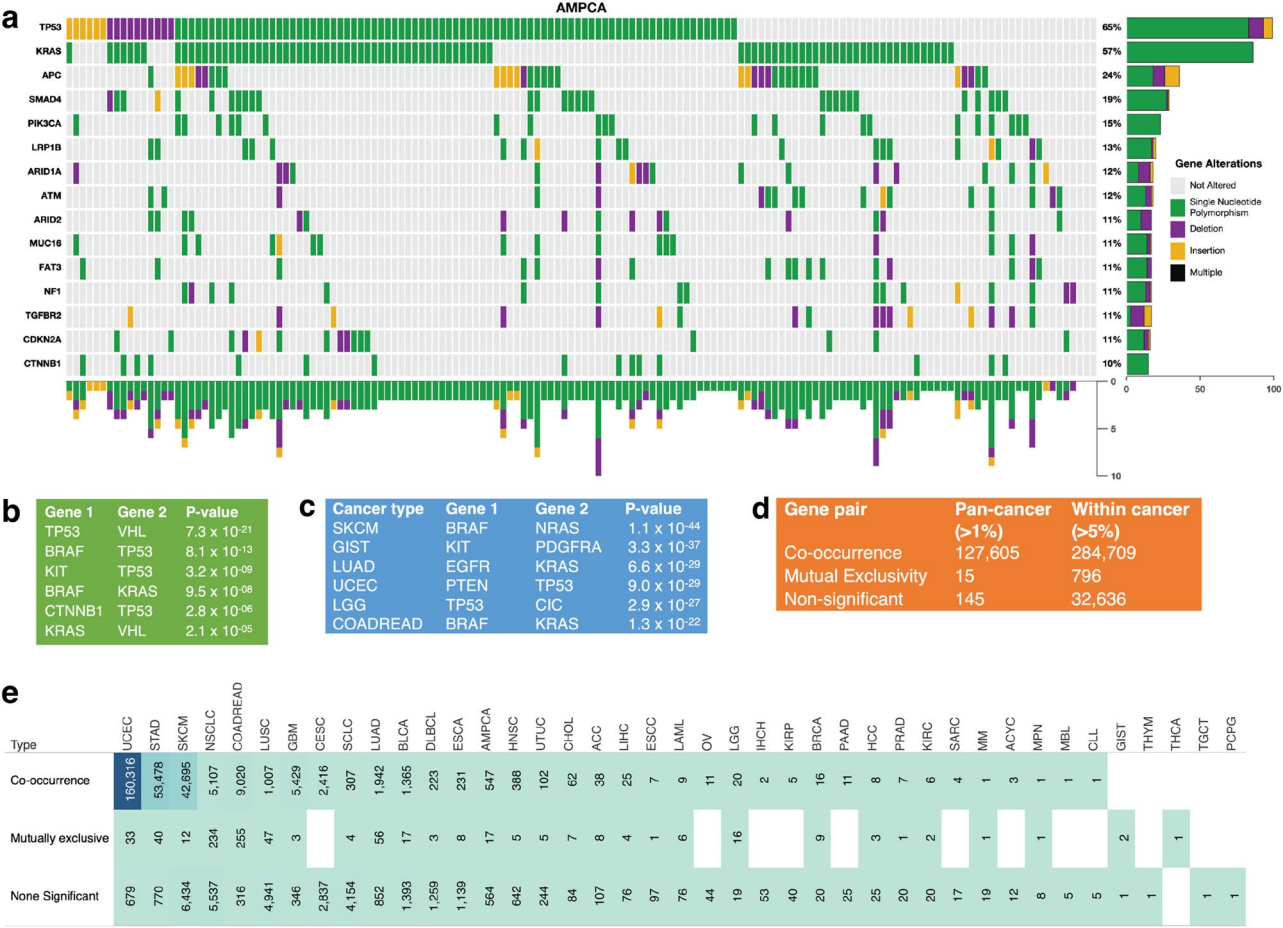
Analysis of co-occurrence and exclusivity of cancer gene mutations across cancer types. (**a**) A plot of non-synonymous somatic mutations in ampullary carcinoma, highlighting the co-occurrence and exclusivity of mutations in the 13 most frequently mutated genes. The plot columns represent samples, and the rows represent mutations. Also, refer to Supplementary Figs. 7a and b for additional information. (**b**) Analysis of co-occurring and exclusive mutations in all cancer types and within each cancer. (**c**) The 7 gene pairs with the highest co-occurrence across pan-cancer studies. (**d**) The 7 gene pairs with the highest mutual exclusiveness within each cancer study. (**e**) Statistics on the number of cancer gene mutations that are co-occurring, mutually exclusive, or neither significantly co-occurring nor mutually exclusive.

Given that we found a convolved pattern in the mutational landscape of the known cancer genes (Fig. 4a and Supplementary Fig. 7a and b), we were interested in determining the extent to which non-synonymous somatic gene mutations tend to be mutually exclusive or co-occur.

First, we assessed mutations in 127,765 gene pairs, both present in at least 1% of 20,331 samples across 41 human cancer types (see the “Methods” section). Here, collectively across all the cancer types, our analysis revealed 127,605 gene pairs with significantly co-occurring mutations, 15 pairs with mutually exclusive mutations, and 145 pairs with non-statistically significant mutations (Fig. 4b). Among the significantly mutually exclusive mutated gene pairs were *VHL* and *TP53* (*p* = 7.3 × 10^–21^), *TP53* and *BRAF* (*p* = 8.0 × 10^–13^), and *BRAF* and *KRAS* (*p* = 9.5 × 10^–8^), see Fig. 4c and Supplementary Data 2. Additionally, the 127,605 significantly co-occurring mutated gene pairs included *PTEN* and *PIK3CA* (*p* = 2.1 × 10^–70^), *BRAF* and *ERBB4* (*p* = 6.9 × 10^–63^), and *EGFR* and *ERBB4* (*p* = 1.9 × 10^–59^), see Supplementary Data 2.

Second, we analysed the mutations in gene pairs that were found to be mutated in more than 5% of the tumours for each of the 41 cancer types. Our results indicate 284,709 gene pairs with significantly co-occurring mutations, 796 gene pairs with significantly mutually exclusive mutations, and 32,636 gene pairs that exhibit a non-statistically significant mutation pattern (see Supplementary Data 2). Furthermore, certain gene pairs were found to exhibit mutually exclusive or co-occurring mutations in specific cancer types. For example, among the top three exclusively mutated gene pairs are *BRAF* and *NRAS* in skin cutaneous melanoma (*p* = 1.1 × 10^–44^), *KIT* and *PDGFRA* in gastrointestinal stromal tumour (*p* = 3.3 × 10^–37^), and *EGFR* and *KRAS* in lung adenocarcinoma (p = 6.6 × 10^–29^) (Fig. 4c). Additionally, we identified specific cancer types in which certain gene pairs exhibit a significantly co-occurring mutation pattern, such as *TP53* and *ATRX* in brain lower-grade glioma (p = 8.2 × 10^–59^), *TP53* and *PTEN* in uterine corpus endometrial carcinoma (p = 9.0 × 10^–29^), *FGFR1OP* and *MAP3K1* in colorectal adenocarcinoma (p = 2.7 × 10^–38^) and uterine corpus endometrial carcinoma (p = 2.9 × 10^–05^). The complete list of mutually exclusive and co-occurring mutated gene pairs in specific cancer types can be found in Supplementary Data 2.

Our analysis of non-synonymous somatic gene mutations in known cancer genes revealed a convolved pattern of mutually exclusive and co-occurring mutations across different human cancer types. Notably, we found more exclusive and co-occurring gene pair mutations within cancer types (796 and 284,709 pairs) than across all types (15 and 127,605 pairs) (Fig. 4d). This result suggests that there may be a selection for specific mutations in certain cancer gene pairs in specific cancer types^29^. Additionally, we propose that the exclusively mutated gene pairs identified in this study may disrupt divergent oncogenic pathways in specific cancer types, providing new insights into the genetic underpinnings of these diseases^30,31^.

Finally, we also analysed gene mutation patterns for each cancer type by aggregating the co-occurring, non-significant, and exclusive mutations per type. For example, as shown in Fig. 4e, colorectal adenocarcinoma had the highest number of exclusively mutated gene pairs among the 41 cancer types, with 255 pairs identified. This was followed by non-small cell lung cancer (234 pairs) and lung adenocarcinoma (55 pairs). On the other hand, uterine corpus endometrial carcinoma exhibited the highest number of co-occurring mutations in gene pairs, with 160,320 pairs identified. This was followed by skin cutaneous melanoma (42,695 pairs) and stomach adenocarcinoma (53,478 pairs), as seen in Supplementary Data 2. These results provide further insight into the distinct genetic profiles of different cancer types and the specific mutations that may drive their development.

### Differences in gene pair co-occurrence and exclusivity among cancer types

**Figure 5:**
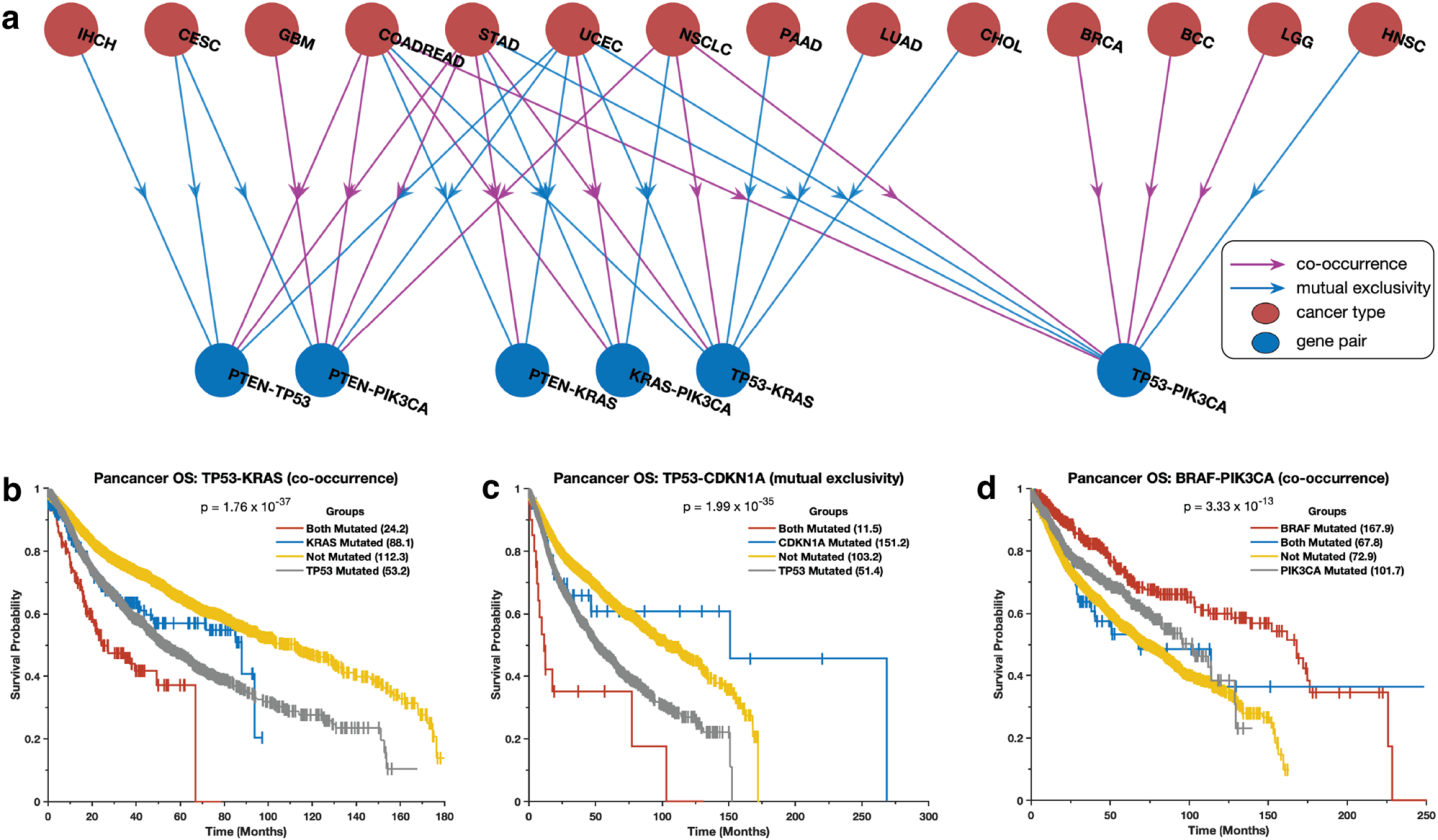
Combination of mutations associated with survival outcomes. (**a**) The correlation between the presence of specific mutations in the most widely studied cancer genes and patient survival rates for different types of cancer. The Kaplan–Meier curve displays the overall survival periods of patients with tumours that have (**b**) both TP53 and KRAS mutations, only TP53 mutations, only KRAS mutations, or no TP53 or KRAS mutations; (**c**) both TP53 and CDKN2A mutations, only TP53 mutations, only CDKN2A mutations, or no TP53 or CDKN2A mutations; (**d**) both PI3KCA and BRAF mutations, only PI3KCA mutations, only BRAF mutations, or no PI3KCA or BRAF mutations.

We investigated the co-occurrence and exclusivity of mutations in the same cancer gene pairs across all cancer types. Interestingly, we found that certain gene pairs exhibit distinct mutation patterns in different cancer types. For instance, we observed that mutations in the *TP53* and *PIK3CA* genes tend to be mutually exclusive in breast carcinoma, colorectal adenocarcinoma, and brain lower-grade glioma, but co-occur in non-small cell lung cancer (Fig. 5a). Additionally, *TP53* and *KRAS* mutations co-occur in lung adenocarcinoma and pancreatic ductal adenocarcinoma but are mutually exclusive in uterine corpus endometrial carcinoma and cholangiocarcinoma (Supplementary Data 2). These observations suggest that when gene pairs are co-mutated, they may work together to promote oncogenesis^32–35^, while when they are exclusively mutated, they may act independently and yield tumours with different phenotypic or molecular subtypes^30,31,36^.

Previous research suggests that there may be a positive correlation between the number of exclusive mutations and co-occurring mutations in a given cancer type, potentially driven by the relationship between mutation burden^37,38^ and the epistatic interaction between driver genes^39,40^. However, the results of the study showed that this was not the case, as there was no correlation between the number of exclusive mutations and co-occurring mutations, nor was there a correlation between the number of cancer-type samples and the number of exclusively mutated gene pairs observed (Supplementary Fig. 8a and b). The lack of correlation was particularly pronounced in uterine corpus endometrial carcinoma, where 160,316 co-occurring gene pairs were found but only 33 exclusive gene pairs across 494 samples. The results suggest that the number of mutually exclusively mutated gene pairs may indicate the genomic complexity of a particular form of cancer and its link to alterations in different oncogenic pathways.

### Disease outcomes of cancer patients are linked to the mutation patterns

We evaluated the impact of mutations in gene pairs on the overall survival of cancer patients by grouping patients into four groups based on the presence of mutations in a gene pair (see the “Methods” section). The groups were: (1) no mutations, (2) and (3) only one of the genes in the pair is mutated, and (4) both genes are mutated. Here, we found that mutations in gene pairs are associated with varied overall survival durations of patients afflicted. For example, in the case of some of the most studied genes in cancer^41,42^, we found that patients with tumours that harbour mutations in both *KRAS* and *TP53* (*p* = 1.76 × 10^–37^) or *CDKN1A* and *TP53* (*p* = 1.99 × 10^–35^) tended to exhibit worse survival outcomes than those with tumours in which one or none of these genes are mutated (Fig. 5b and 5c, see Supplementary Data 3). Furthermore, we found that patients with tumours with mutations in *PIK3CA* and/or *BRAF* tended to exhibit better survival outcomes than those with mutations in *TP53*, *KRAS*, and/or *EGFR* (Fig. 5d and Supplementary Fig. 9). In addition, we found that the patients with tumours with mutations in *PIK3CA*, *BRAF*, *CDH1*, and *NRAS* exhibit better survival outcomes than those without mutations in these genes (Supplementary Fig. 9; and Supplementary Data 3).

Our findings highlight the importance of understanding the impact of different combinations of gene mutations on cancer development and progression. Specifically, some cancer patients may exhibit significantly different disease outcomes due to the specific combination of mutations present in their tumours, as has been demonstrated in multiple studies^43–45^. For example, mutations in the KRAS that co-occur with STK11, KEAP1, and TP53 genes in lung cancer patients have been associated with poorer prognosis and reduced survival^44^.

### Driver pathways of co-occurring and exclusively mutated genes

**Figure 6:**
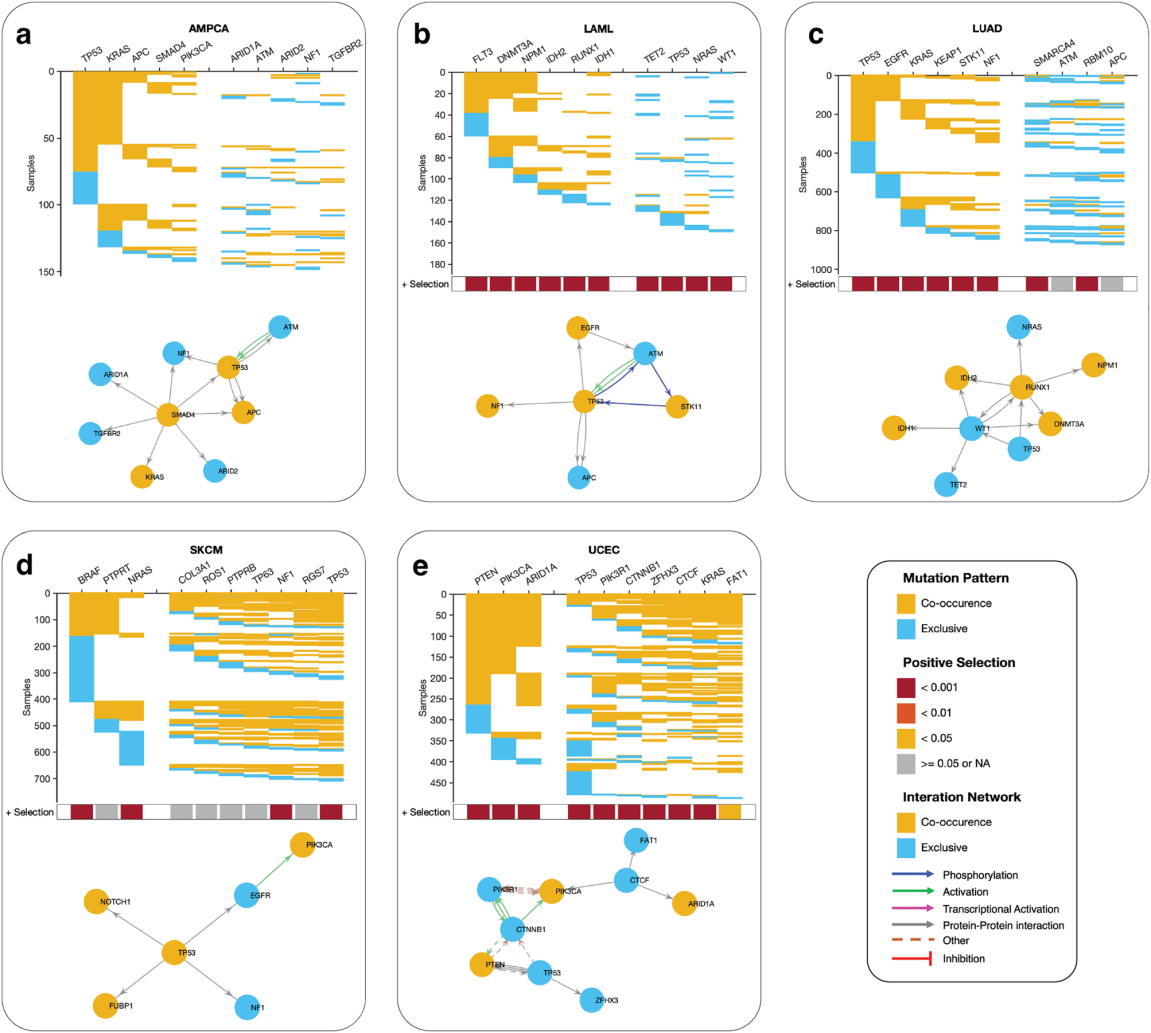
Predictive oncogenic pathways in different cancer types based on mutational landscapes. Two co-occurring pathways are predicted to drive oncogenesis based on the mutational landscape in (**a**) AMPCA, (**b**) LAML, (**c**) LUAD, (**d**) SKCM, and (**e**) ECEC. The coloured square marks at the bottom of each plot show a positive selection of mutations in each gene along each column (see the “Methods” section). The connectivity of network components within each panel was extracted from the KEA and ChEA databases and the UCSC super pathway.

Our study sought to identify the driver pathways (gene combinations) for the top 10 most frequently mutated cancer genes in various cancer types. Using a detailed analysis of gene pairs, we found intriguing variations in patterns of co-occurring and mutually exclusive mutations across different cancer types (see “Methods” sections and Supplementary Data 4). In ampullary carcinoma, for example, we identified two sets of co-occurring mutated driver pathways (Fig. 6a). The first set involves five genes (*TP53*, *KRAS*, *APC*, *SMAD4*, and *PIK3CA*) that exhibit a co-occurring mutation pattern, while the second set of five genes (*ARID1A*, *ATM*, *ARID3*, *NF1*, and *TGFBR2*) are exclusively mutated. Similarly, in acute myeloid leukaemia, we found two gene sets; the first set includes six genes (*FLT3, DNMT3A, NPM1, IDH3, RUNX1,* and *IDH1*) that exhibit a co-occurring mutation pattern, and the second set of four genes (*TET2, TP53, NRAS,* and *WT1*), exhibit an exclusive mutation pattern (Fig. 6b). In lung adenocarcinoma, we found two gene sets; the first set includes six genes (*TP53, EGFR, KRAS, KEAP1, STK11,* and *NF1*) that exhibit a co-occurring mutation pattern, and the second set of four genes (*SMARCA4, ATM, RBM10,* and *APC*) (Fig. 6c), exhibit an exclusive mutation pattern.

However, our analysis revealed that driver gene mutations have a high co-occurrence in specific cancer types, including uterine corpus endometrial carcinoma, skin cutaneous melanoma, and basal cell carcinoma (Fig. 6d,e, Supplementary Fig. 10). We also observed that the mutated genes of cancer driver pathways are significantly under positive selection across all cancer types, highlighting the potential importance of these genes in cancer development and progression.

Here, our findings suggest that different cancer types may exhibit distinct patterns of driver pathway mutations, and that further research is needed to fully understand the implications of these patterns for cancer subtypes and cancer treatment.

### Cancer gene mutations in the context of cancer hallmarks

**Figure 7:**
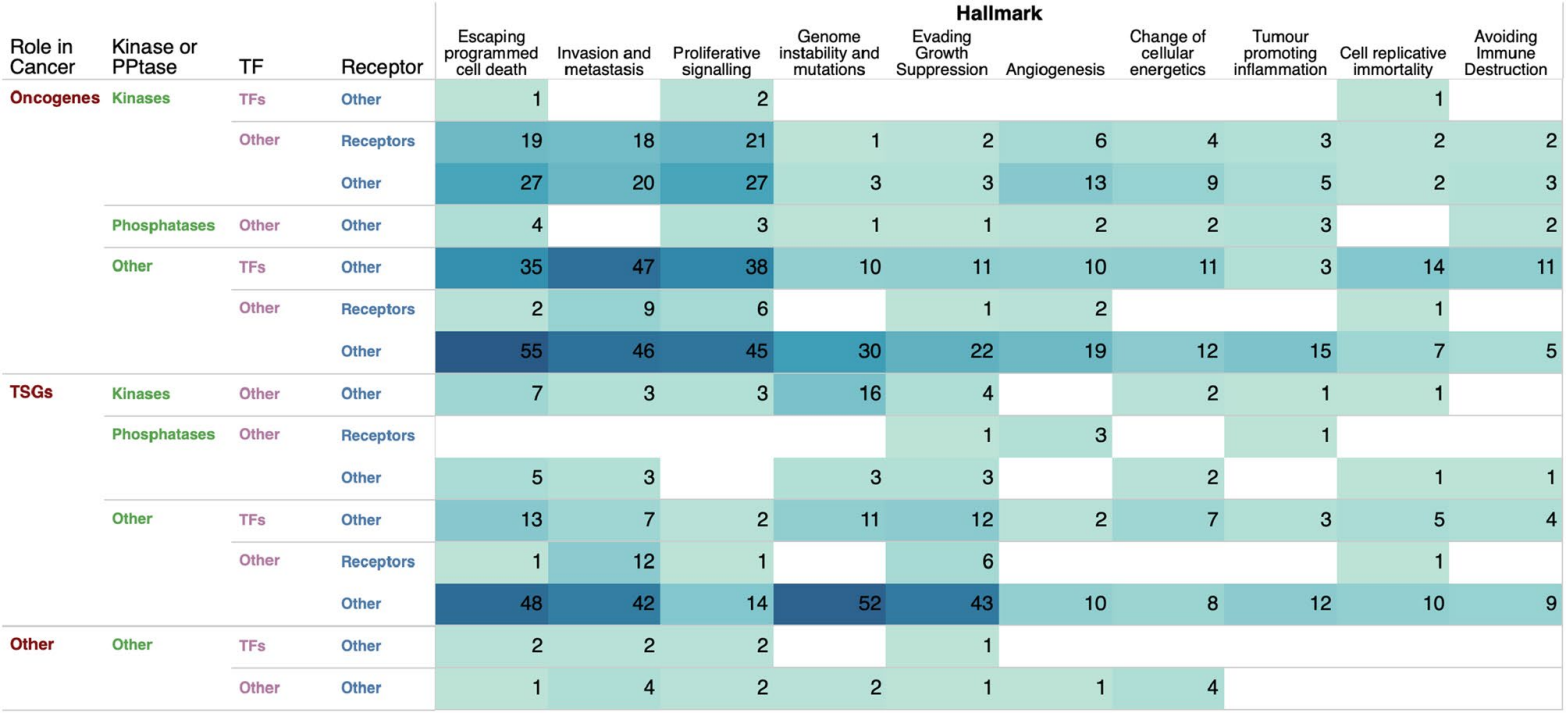
Alterations of cancer hallmark genes. The total number of cancer genes across each combination of cancer genes categories associated with the hallmarks of cancer.

Owing to the importance of cancer hallmarks in designing better treatment strategies, we sought to determine the extent to which genes associated with each hallmark of cancer are altered across different types of human cancer (Fig. 7 and Supplementary Data 5, see "Methods" section). Our analysis revealed that the highest number of mutated genes were found in the "escaping programmed cell death" hallmark (220 genes), followed by "invasion and metastasis" (213 genes), "proliferative signalling" (160 genes), and "genome instability and mutations" (129 genes), as shown in Fig. 7. Notably, the most frequently mutated genes within these hallmarks were oncogenes and tumour suppressor genes that are not kinases, phosphatases, or cell surface receptors. This is of particular interest as current efforts in cancer research to identify drug targets primarily focus on kinases and cell surface receptors. Our findings suggest the potential for identifying a diverse range of drug targets among non-traditional cancer gene targets.

## Discussion

In this study, we have conducted a systematic analysis of 727 cancer gene mutations across 41 human cancer types. Our results reveal the presence of non-synonymous mutations in known cancer genes in all samples examined, including mutations in oncogenes, TSGs, genes encoding transcription factors, kinases, phosphatases, and cell surface receptors. This suggests that various components of the cell signalling process are involved in oncogenesis. Furthermore, this finding demonstrates that various components of the cell signalling processes, including receptors that respond to stimuli, cytoplasmic enzymes, and nuclear proteins, are involved in oncogenesis. Interestingly, we found that not all samples of a particular cancer type harbour the same driver mutations, and the distribution of gene mutations within each cancer type varies significantly. These findings suggest that each patient may exhibit a different combination of mutations sufficient to perturb various oncogenic pathways, highlighting the importance of understanding the mutation profile of each patient's tumour for the optimization of personalised cancer treatments.

In recent years, there has been a growing understanding of the importance of identifying driver mutations in different cancer types^3,8,10,31^. Studies have identified specific driver mutations in various cancers, such as EGFR mutations in lung cancer^46^ and BRAF mutations in melanoma^47^. These findings have led to the development of targeted therapies that specifically target the mutated genes or pathways, improving patient survival rates and quality of life^48–51^. For example, the discovery of EGFR mutations in lung cancer led to the development of targeted therapies such as gefitinib and erlotinib, which have demonstrated improved outcomes compared to traditional chemotherapy^52,53^. Similarly, the identification of BRAF mutations in melanoma led to the development of targeted therapies such as vemurafenib and dabrafenib, which have also improved patient outcomes^54,55^. Therefore, we suggest that a deeper understanding of the interplay between driver mutations in cancer can lead to more effective and personalised treatments for different cancer types and subtypes that reduce the burden of cancer and improve patient outcomes.

However, it is important to note that some tumours have fewer than 5% of their cancer genes mutated. For example, gene mutations are infrequent in thyroid carcinoma, testicular germ cell tumours, and thymomas, where only two cancer genes are mutated in more than 5% of the examined tumours. These exceptions reinforce the notion that multiple routes to oncogenesis may be independent of cancer gene mutations and involve alterations in other regulatory mechanisms, such as the epigenome^1,7,56^. Furthermore, these findings reveal that in cases where cancer gene mutations are infrequent, other genetic datasets, such as chromosomal changes, epigenetic changes, copy number variations, microsatellite instability, mRNA transcription abundance, and mutations in the non-coding and regulatory regions of the genome, should be considered to identify cancer genes^57–61^.

Despite the large amounts of genomic data this study analysed, we could not pinpoint commonly mutated cancer genes in samples of specific cancer types. This highlights the sparsity nature of gene mutations and the limited diversity of the presently available genome sequences, which impede the identification of commonly applicable drug targets and marker mutations within each cancer type^62–66^. Despite this, we discovered that in gene pairs specific to all types of cancer, the co-occurrence of mutations (284,709) is 378 times more likely than exclusive mutations (796). This demonstrates that genes involved in various cancer pathways have a greater tendency to co-mutate rather than mutate exclusively^40,67^. As a result, our findings suggest the presence of a limited number of context-dependent, co-occurring driver gene mutations, which could facilitate the identification of widely applicable drug targets and markers of aggressiveness across a multitude of patients' tumours.

Our findings also show that the co-occurrence and exclusive nature of cancer gene mutations significantly affect the disease outcome of patients with various forms of cancer. In addition, these results indicate that various gene alterations in specific gene pairs have a diverse impact on processes that drive disease aggressiveness^68–70^.

However, a limitation of our study is that we did not account for the distinction between COSMIC Tier 1 and Tier 2 cancer-driving genes in our analysis^23^. Tier 1 genes have well-documented activities relevant to cancer, while Tier 2 genes have strong indications but with emerging evidence^23^. By not distinguishing between these tiers, we may have overlooked some nuanced differences in the role these genes play in oncogenesis. This could potentially affect the interpretation of the data, especially in the context of clinical relevance. In future studies, a separate analysis of Tier 1 and Tier 2 genes might provide more detailed insights into their distinct roles and contributions to the development and progression of cancer.

In conclusion, to comprehensively evaluate the impact of different combinations of gene alterations on cancer development and treatment response, there is an urgent need for new molecular tools. While there is a vast amount of genomics data available, it is currently not feasible to study the impact of every possible combination of gene mutations due to the sheer size of the combinatorial space. It is important to note that the currently available genomics data is limited to a subset of patient tumours and cell lines, which only includes a fraction of the possible combinations of driver mutations that may exist. While databases^15,23,42^ and computational tools can be used to predict the potential impact of certain gene mutations or their combinations^71–73^, the limitations of available data and the complexity of cancer genetics make it challenging to comprehensively evaluate the impact of every possible combination on cancer development and drug response. As such, future experiments that allow the altering of cancer genes in normal cells in different combinations will ultimately help to unlock the impact of a combination of cancer gene mutations on oncogenesis, disease aggressiveness, and the chemosensitivity of tumours. By developing new molecular tools and continuing to expand our knowledge of cancer genetics, we can move closer to achieving precision medicine in cancer treatment, where patients receive tailored therapies based on the genetic profile of their cancer.

## Methods

In our study, we obtained a dataset of 366 cancer studies from the cBioPortal^74^ version 5.2.5 (http://www.cbioportal.org) of deeply sequenced tumours. To ensure a high level of specificity, we excluded studies that employed targeted sequencing, datasets of paediatric tumours, and cancer cell lines, resulting in a final dataset of 206 cancer studies. Further, we filtered the dataset again to return only those from 138 cancer studies that also had clinical information for the profiled afflicted patients. The final datasets encompassed 20,331 patient-derived tumour samples representing 41 distinct human cancer types sequenced from the year 2012 to 2021 (see Supplementary Data 1 for details on the cancer studies). The elements of the data that we obtained from cBioPortal include somatic gene mutations (point mutations and small insertions/deletions) and comprehensively deidentified clinical data.

### Compilation of cancer genes and their classes

In our study, we sourced data on cancer-driving genes the Catalogue of Somatic Mutations in Cancer^24^ (COSMIC), specifically from the Consensus Cancer Gene Database^23^ (version 95, released on November 24, 2021), which includes a total of 727 cancer-driving genes that have been rigorously vetted for evidence and manually curated, with 576 in Tier 1 and 151 in Tier 2. To further classify these known cancer genes, we divided them into five classes: oncogenes, tumour suppressor genes, kinases, phosphatases, cell surface proteins, and transcription factors. To achieve this, we obtained information on gene annotations from various databases, including the Sanger Consensus Cancer Gene Database^23^ (699 oncogenes and TSGs), the UniProt Knowledgebase^75^ (304 oncogenes and 741 TSGs), the TSGene database^76^ (1,220 TSGs), the ChEA transcription factor database^77^ (645 transcription factors), the TF2DNA database^78^ (1,314 transcription factors), the Kinase Enrichment Analysis database^79^ (428 kinases), the ONGene database^80^ (725 oncogenes), and the Surfaceome database^81^ (2,950 cell surface receptors). We then collated the cancer genes from all the above databases to obtain a list of 727 cancer genes (after removing overlapping genes), including 383 oncogenes, 370 tumour suppressor genes, 9 phosphatases, 75 kinases, 63 cell surface receptors, and 150 transcription factors (Supplementary Data 1).

### Calculation of cancer gene mutations

We obtained the gene sequencing datasets of the samples for all the cancer genes. We then selected only the non-synonymous mutations that occurred within the genes. To evaluate the extent to which each cancer-driving gene is mutated in cancer, we calculated the somatic mutation frequency (including single nucleotide mutations, short indels, and insertions) for each gene across the 20,331 samples across each cancer type (Supplementary Data 1). Additionally, for each cancer type, we obtained summaries of the number of mutated genes in (1) none of the samples, (2) less than 5% of the samples, and (3) more than 5% of the samples.

### Cancer gene mutations across gene classes

We aimed to determine the extent of mutations in cancer-associated genes within individual cancer types and across all human cancers. To achieve this, we calculated the non-synonymous somatic mutation frequency (including single nucleotide mutations, short indels, and insertions) for each of the 41 human cancer types represented among the 20,331 samples (Supplementary Data 1). Additionally, we calculated the frequency of non-synonymous somatic mutations for groups of genes, such as oncogenes, tumour suppressor genes, kinases, phosphatases, cell surface receptors, and transcription factors, across each of the 41 cancer types and the entire patient cohort (Supplementary Data 1). It should be noted that, for the calculations involving gene categories, we included genes that belong to each category and not only exclusively to one category.

### Assessment of the co-occurrence and exclusivity of gene mutations

We used the hypergeometric Fisher test to evaluate the correlation in the mutation profile of cancer gene pairs. First, we obtained a list of mutated genes in more than 1% (550 cancer genes) of all tumours across all the samples. Next, we applied the Fisher test to each pair of the selected genes and utilised a cut-off p-value of 0.05 to identify statistically significant gene pair correlations. Furthermore, we used the magnitude of the odds ratio to identify gene pairs with co-occurring mutations (odds > 1 and p < 0.05) and gene pairs with mutually exclusive mutations (odds < 1 and p < 0.05). Additionally, we used the approach to identify cancer gene pairs within co-occurring or mutually exclusive mutation patterns within each of the 43 human cancer types (see Supplementary Data 2).

### Correlation between mutations pattern and disease outcomes

We used the Kaplan–Meier^82^ method to estimate the duration of overall survival and disease-free survival of patients with cancer who had (1) only one gene pair mutated, (2) the other gene pair mutated, (3) none of the gene pair mutated, and (4) both gene pairs mutated.

### Identification of exclusive and co-occurring driver pathways

To identify the patterns of mutations associated with each cancer type and the corresponding cancer gene combinations, we applied the CoMDP algorithm^83^. This algorithm employs a mathematical programming method to identify de novo driver pathways in cancer from mutation profiles. Specifically, we aimed to identify pathways with mutated cancer genes that exhibit both high coverage (i.e., present in multiple samples) and high exclusivity, and show a statistically significant co-occurrence pattern.

To begin, we selected the top 10 mutated cancer genes for each cancer type as input for the CoMDP algorithm. These genes were selected from the significantly mutated genes identified using the MutSigCV2 algorithm^84^. In cases where MutSigCV2 identified fewer than 10 genes as significantly mutated, we included additional genes to bring the gene set size to 10. If a gene with very long coding regions or very long introns (including *CSMD1, CSMD3, NRXN1, NRXN4, CNTNAP2, CNTNAP4, CNTNAP5, CNTN5, PARK2, LRP1B, PCLO, MUC16, MUC4, KMT2C, KMT2A, KMT2D, FAT1, FAT2, FAT3,* and *FAT4*) were not identified as being significantly mutated using MutSigCV2 in a specific cancer type, it was excluded from the analysis for that cancer type^84^.

Next, we ran the CoMDP test for each cancer type with K = 10, where K equals the gene set size. The CoMDP analysis returned mutated driver pathways associated with the genes in each cancer type (Supplementary Data 4). Additionally, we obtained information on the selectivity of gene mutations in each cancer type from the supplementary data of Martincorena et al.^29^.

### Association between cancer gene mutations and the cancer hallmarks

To investigate the relationship between the Hallmarks of Cancer^1,85^ and the mutated gene sets, we accessed information on genes and proteins associated with various Hallmarks of Cancer from the COSMIC database^24^. We then calculated the number of mutated genes at a frequency of more than 1% in at least one cancer type within each class based on the various cancer hallmark gene sets (Supplementary Data 5).

### Statistics and reproducibility

We used MATLAB 2022a^86^ for all statistical analyses. We used two-sided statistical tests with a *p*-value < 0.05 to indicate statistical significance. To correct for multiple statistical testing, we applied the Benjamini & Hochberg procedure^87^, resulting in a q-value < 0.05 for each comparison.

### Ethics approval

The study protocol was approved by The University of Cape Town; Health Sciences Research Ethics Committee IRB00001938. The publicly available datasets were collected by the cBioPortal.

### Supplementary Information

Supplementary Legends.

Supplementary Information 1.

Supplementary Information 2.

Supplementary Information 3.

Supplementary Information 4.

Supplementary Information 5.

Supplementary Figures.

## Data Availability

The data that support our results are available in this manuscript, the supplementary data, and from the following repositories: cBioPortal; https://www.cbioportal.org/, and the COSMIC Consensus Cancer Genes; https://cancer.sanger.ac.uk/census.

## Code availability

Custom code written in MATLAB for processing and analysing the presented data is freely available at https://github.com/smsinks/Analysis-of-Cancer-Gene-Mutations. In addition, the repository includes some pre-downloaded datasets and conversion files required for the analysis.
