# Supplementary material for: Mutational landscape of cancer-driver genes across human cancers
Source: Sci Rep. 2023 Aug 7;13:12742. doi: 10.1038/s41598-023-39608-2 (PMC10406856; doi:10.1038/s41598-023-39608-2)
Supplement: Supplementary file 1 — Supplementary Legends. [file 41598_2023_39608_MOESM1_ESM.pdf]

Mutational Landscape of Cancer-Driver Genes Across Human Cancers

Description of Additional Supplementary Files

Supplementary Data 1: Cancer studies and mutations in cancer genes. The spreadsheet includes the following information, organized by sheet name:

- Cancer Genes: A list of well-known cancer driver genes obtained from the COSMIC database[1], including the official HUGO gene symbol and the class of protein encoded by the genes.
- Cancer Studies: A list and description of individual cancer studies used in our analysis.
- Cancer Mutations: The frequency of mutations and the percentage of tumours that contain mutations in each known cancer gene.
- OGs and TSGs: Mutations found in oncogenes and tumour suppressor genes across all cancer studies.
- Kinases and Phosphatases: Mutations found in kinases and phosphatases across all cancer studies.
- Transcription Factors: Mutations found in transcription factors across all cancer studies.
- Each Gene Mutation: Overall mutation frequencies across all cancer types for each gene.
- Mutation Freq Grouped: Frequency of gene mutations across cancer types, related to Supplementary Figure 1.

Supplementary Data 2: The spreadsheet contains information on gene mutations in cancer, categorized according to sheet name:

- Across cancer Exclusive: lists gene pairs significantly mutated across all cancer types.
- Across Cancer Mutations: shows all cancer gene mutations across all cancer types. Each Gene Pair in Cancer Type: details the mutation pattern of each gene pair in each cancer type.
- Summary of Mutation Pattern: summarizes the number of mutually exclusive, co-occurring, and non-significantly mutated genes in each cancer type.
- Within cancer Exclusive: lists gene pairs that are significantly exclusively mutated within each cancer type.

Supplementary Data 3: The spreadsheet contains clinical outcome information calculated using the Log-rank test [2], categorized according to sheet name:

- PanCancer OS: shows overall survival for patients with tumours grouped based on gene pair mutations (no mutations, one mutated gene, or both mutated).
- PanCancer MultipCompare OS: compares the overall survival periods between patient groups.
- PanCancer DSF: shows disease-free survival for patients with tumours grouped based on gene pair mutations.

- PanCancer MultiCompare DFS: compares disease-free survival periods between patient groups.

Supplementary Data 4: The spreadsheet shows co-occurring gene sets mutated in each cancer type:

- Gene sets 1 and 2: represent co-occurring driver pathways in each cancer type.
- n1 and n2: denote the coverage of genomic alterations in each cancer type.
- r1,2: is the ratio of common coverage to union coverage (i.e., co-occurrence ratio). Co-occurrence P: represents the p-value of co-occurrence significance.

Supplementary Data 5: The spreadsheet shows mutations of cancer driver genes across various cancer hallmarks and is related to Figure 7.

References

1. Sondka, Z. *et al.* The COSMIC Cancer Gene Census: describing genetic dysfunction across all human cancers. *Nat. Rev. Cancer 2018 1811* **18**, 696–705 (2018).
2. Goel, M. K., Khanna, P. & Kishore, J. Understanding survival analysis: Kaplan-Meier estimate. *Int. J. Ayurveda Res.* **1**, 274–8 (2010).
