# Supplementary material for: Mutational landscape of cancer-driver genes across human cancers
Source: Sci Rep. 2023 Aug 7;13:12742. doi: 10.1038/s41598-023-39608-2 (PMC10406856; doi:10.1038/s41598-023-39608-2)
Supplement: Supplementary file 7 — Supplementary Figures. [file 41598_2023_39608_MOESM7_ESM.pdf]

Mutational Landscape of Cancer-Driver Genes Across Human Cancers

Supplemental Information

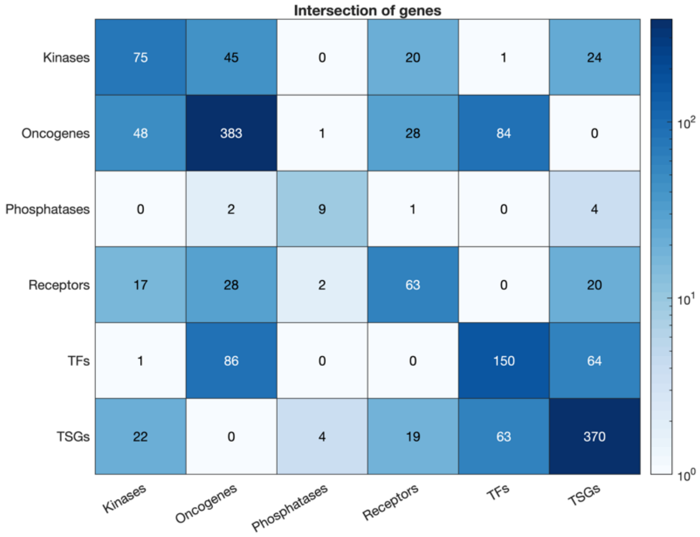

Supplementary Figure 1: *Categories of known cancer genes with the main diagonal of the heatmap indicating the total gene count in each category. Off-diagonal numbers show the number of shared genes between the categories indicated by the rows and columns.*

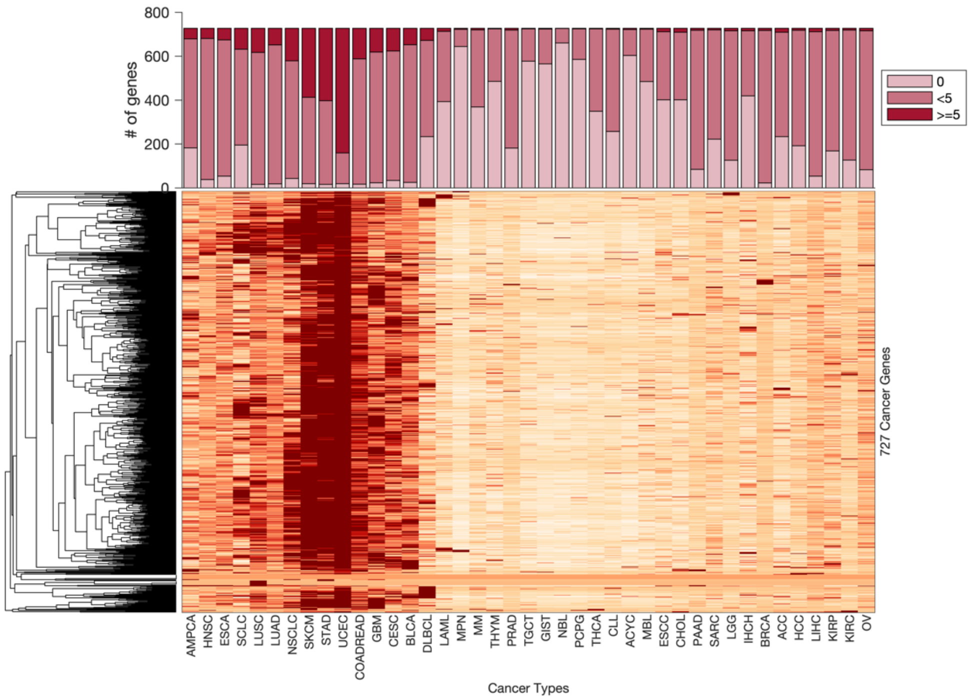

Supplementary Figure 2: *Unsupervised hierarchical clustering of tumours based on mutation frequency of driver genes, with a grouped bar graph at the top indicating the number of driver genes mutated in >=5% (dark), <5% (intermediate), or 0% (light) of the samples.*

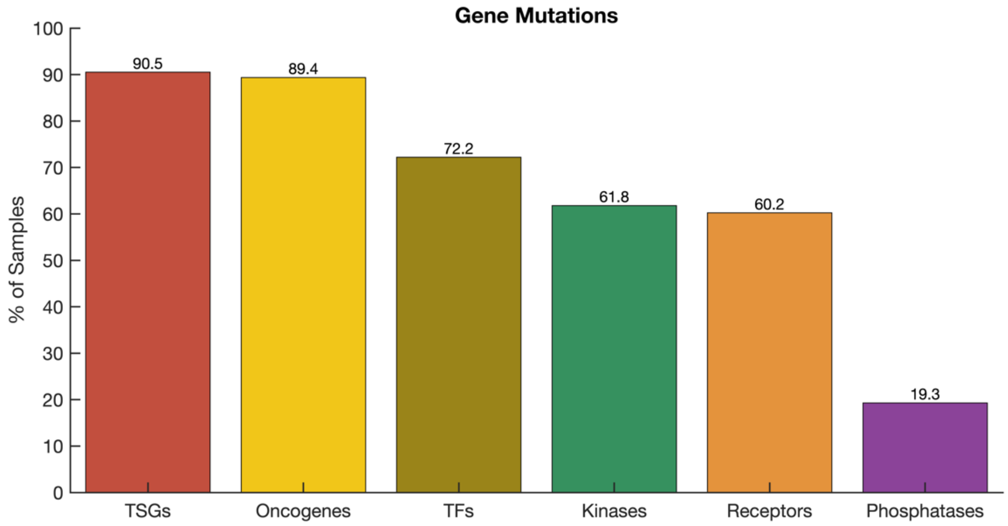

Supplementary Figure 3: *Frequency of driver genes from various classes across all tumour samples.*

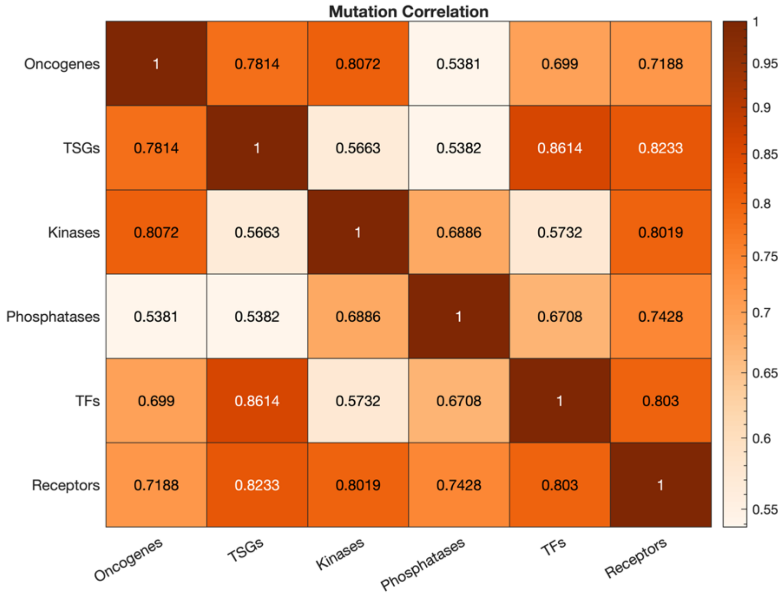

Supplementary Figure 4: *Correlation of mutation signature of cancer gene categories across 41 cancers. Heatmap numbers indicate Pearson's correlation scores, with increasing color intensity showing higher correlation.*

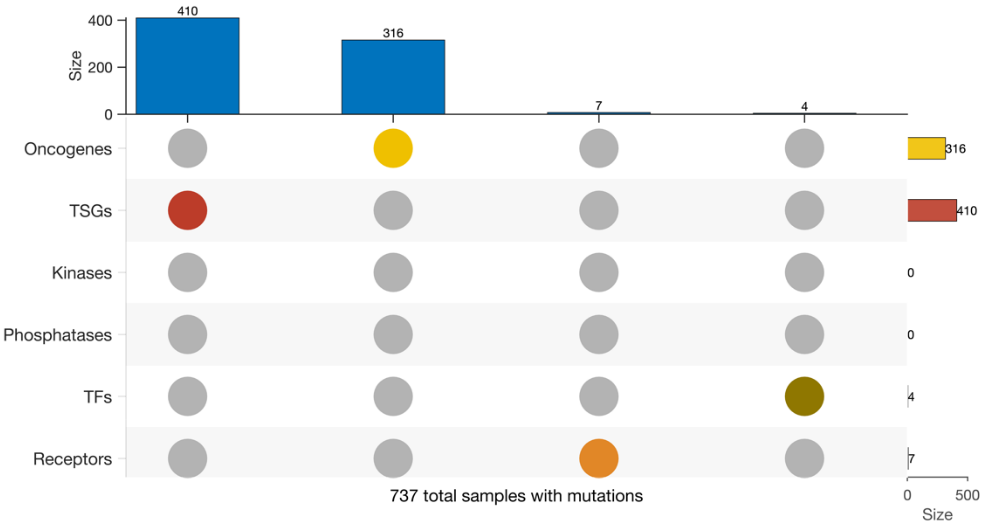

Supplementary Figure 5: *Upset plot of the count of mutually exclusive mutated driver genes in all tumour samples.*

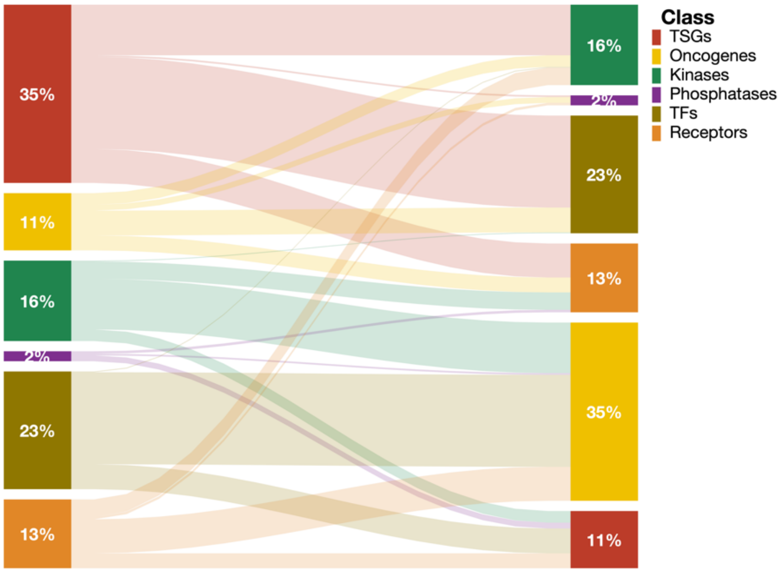

Supplementary Figure 6: *Percentage of mutated cancer genes belonging to multiple categories. Note, the plotted data includes on the genes that have multiple cancer gene categories.*

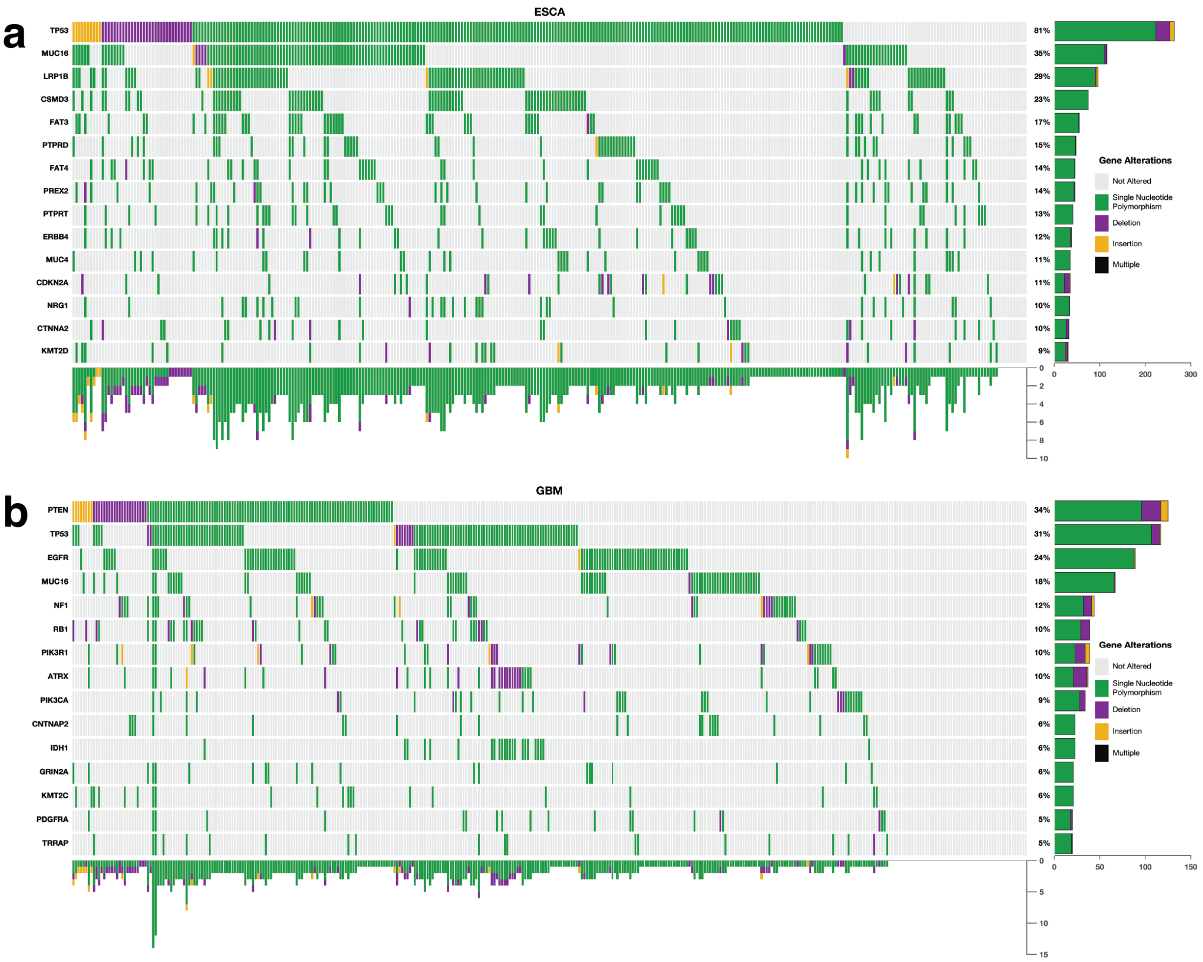

Supplementary Figure 7: *Example of mutation signature plot with co-occurrence and exclusivity of mutations in oesophageal (a) and kidney (b) cancer, displaying only the 15 most mutated genes.*

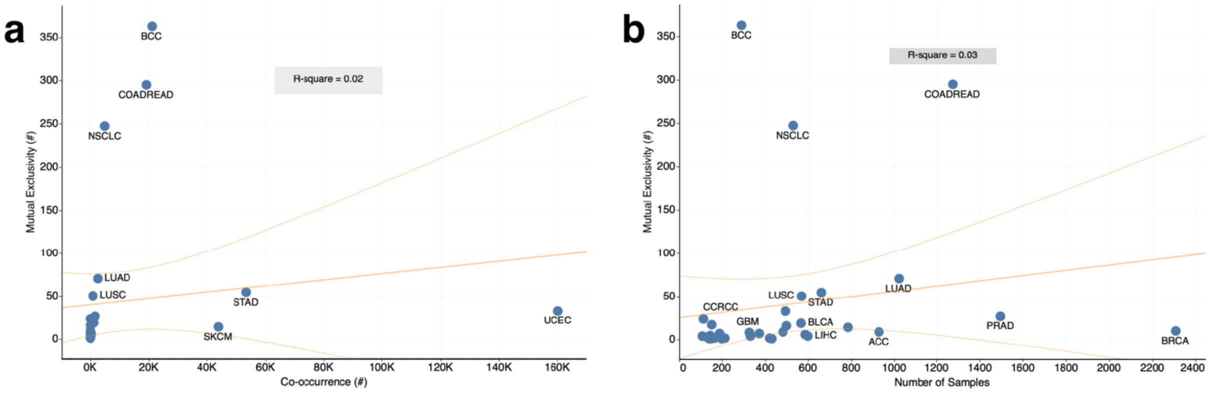

Supplementary Figure 8: *Correlation between the number of co-occurring (a) and exclusive (b) mutations in gene pairs vs the number of profiled samples in each cancer type.*

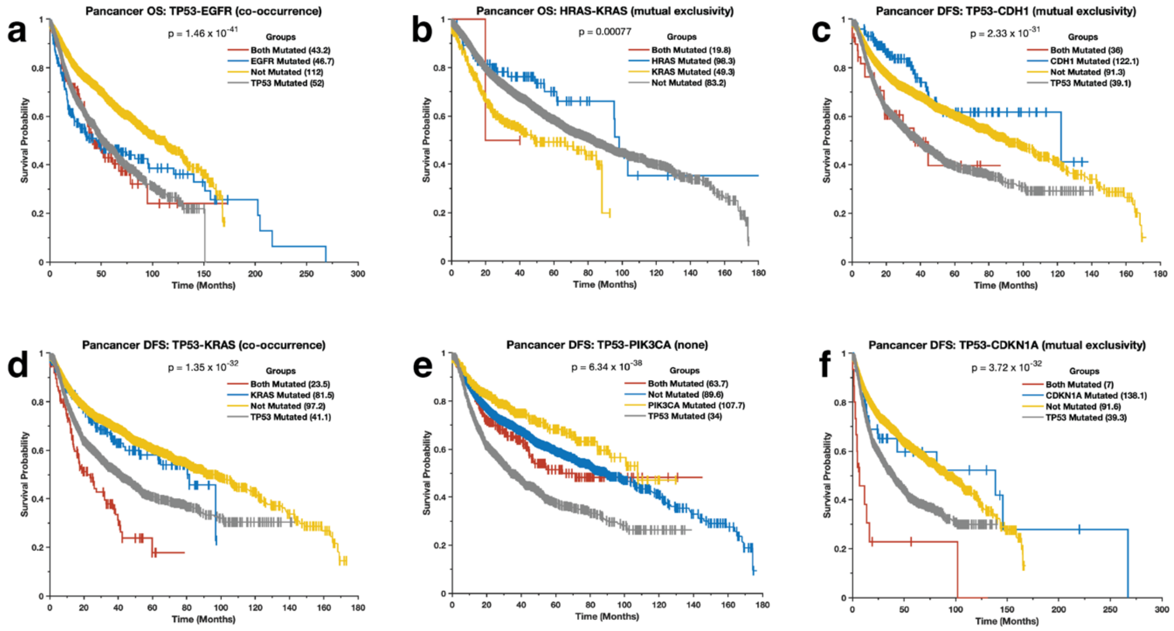

Supplementary Figure 9: Kaplan-Meier curve showing disease-free and overall survival of patients with tumours that have two mutated driver gene pairs, one mutated gene, or no mutated genes in the pair.

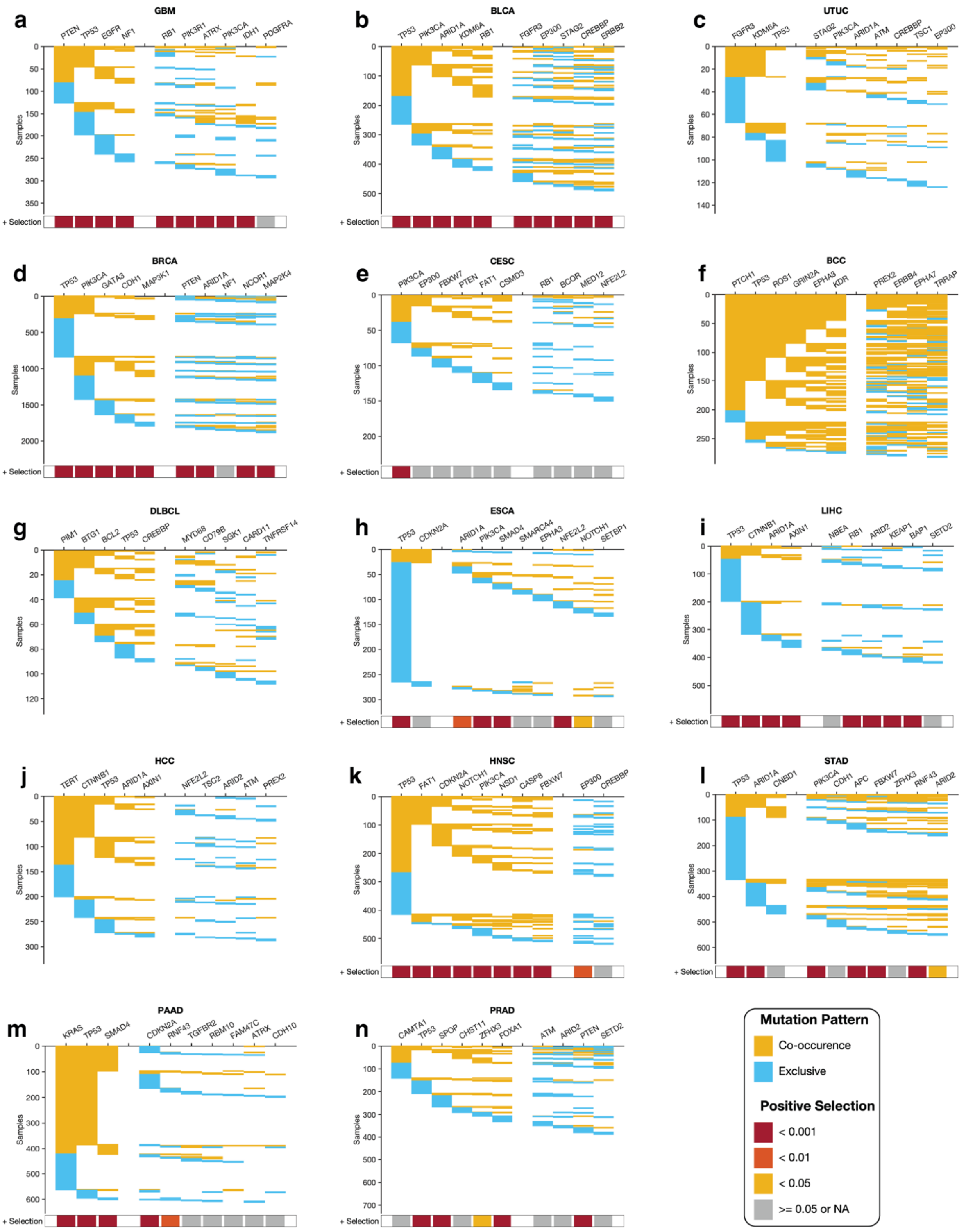

Supplementary Figure 10: *Two co-occurring pathways driving oncogenesis based on mutational landscape in various cancers, with coloured squares at the bottom indicating positive selection of mutations in each gene (see Methods section). The connectivity of network components within each panel was extracted from the KEA and ChEA databases and the UCSC super pathway.*
